# Comparison of general anesthesia with endotracheal intubation, combined spinal-epidural anesthesia, and general anesthesia with laryngeal mask airway and nerve block for intertrochanteric fracture surgeries in elderly patients: a retrospective cohort study

**DOI:** 10.1186/s12871-019-0908-2

**Published:** 2019-12-17

**Authors:** Yang Liu, Mang Su, Wei Li, Hao Yuan, Cheng Yang

**Affiliations:** 1 Department of Orthopedics, Chengdu Aerospace Hospital, Chengdu, 610100 China; 2 Department of Anesthesia, Chengdu Aerospace Hospital, Chengdu, 610100 China

**Keywords:** Anesthesia, elderly patient, intertrochanteric fracture, laryngeal mask airway

## Abstract

**Background:**

There is no consensus on the optimal anesthesia method for intertrochanteric fracture surgeries in elderly patients. Our study aimed to compare the hemodynamics and perioperative outcomes of general anesthesia with endotracheal intubation, combined spinal-epidural anesthesia, and general anesthesia with laryngeal mask airway (LMA) and nerve block for intertrochanteric fracture surgeries in elderly patients.

**Methods:**

This is a retrospective study of 75 patients aged > 60 years scheduled for intertrochanteric fracture surgeries with general anesthesia with intubation (*n* = 25), combined spinal-epidural anesthesia (*n* = 25), and general anesthesia with LMA and nerve block (*n* = 25). The intraoperative hemodynamics were recorded, and the maximum variation rate was calculated. Postoperative analgesic effect was evaluated using the visual analog scale (VAS). Postoperative cognitive status was assessed using the Mini-Mental State Exam (MMSE).

**Results:**

The maximum variation rate of intraoperative heart rate, systolic blood pressure, diastolic blood pressure differed significantly between the three groups (general anesthesia with intubation > combined spinal-epidural anesthesia > general anesthesia with LMA and nerve block). The VAS scores postoperative 2 h, 4 h, 6 h, and 8 h also differed significantly between the three groups (general anesthesia with intubation > combined spinal-epidural anesthesia > general anesthesia with LMA and nerve block). The VAS scores postoperative 24 h were significantly lower in the general anesthesia with LMA/nerve block group than the general anesthesia with intubation group and the combined spinal-epidural anesthesia group. The MMSE scores postoperative 15 min and 45 min differed significantly between the three groups (general anesthesia with intubation < combined spinal-epidural anesthesia < general anesthesia with LMA and nerve block). The MMSE scores postoperative 120 min in the general anesthesia with intubation group were the lowest among the three groups. There was no significant difference in the incidence of respiratory infection postoperative 24 h, 48 h, and 72 h between the three groups.

**Conclusion:**

Compared to general anesthesia with intubation and combined spinal-epidural anesthesia, general anesthesia with LMA and nerve block had better postoperative analgesic effect and less disturbances on intraoperative hemodynamics and postoperative cognition for elderly patients undergoing intertrochanteric fracture surgeries.

## Background

The global population aging has led to increasingly more elderly patients with hip fractures. Aging is associated with decreased hemodynamic stability, hypertension, poor physical status, risk of cognitive impairment, and osteoporosis [1–3]. Providing anesthesia for hip fracture surgeries in elderly patients can be challenging.

It has been shown that regional anesthesia is comparable to general anesthesia for hip fractures in adults [4]. However, it is not known whether regional anesthesia is superior to general anesthesia in terms of hemodynamic stability and postoperative cognitive impairment in elderly patients undergoing hip surgeries. A previous study found that general anesthesia with endotracheal intubation was associated with intraoperative hypotension and hemodynamic instability in elderly patients undergoing hip surgeries compared to general anesthesia with laryngeal mask airway (LMA) and nerve block [5]. The effect of these two anesthetic methods on postoperative cognitive function in elderly patients is still not clear.

Our study aimed to compare intraoperative hemodynamics, postoperative pain, postoperative cognitive function, and respiratory infection between general anesthesia with endotracheal intubation, combined spinal-epidural anesthesia, and general anesthesia with LMA and nerve block for elderly patients undergoing intertrochanteric fracture surgeries.

## Methods

### Patients

A total of 75 patients scheduled for closed reduction or open reduction with internal fixation for intertrochanteric fracture between January 2017 and November 2018 were included in this retrospective study. The inclusion criteria were age > 60 years and < 90 years, intertrochanteric fracture diagnosed by radiology, and American Society of Anesthesiologists (ASA) physical status I–III. Patients with the following conditions were excluded: cognitive impairment, allergy to anesthetic agents, operation time > 4 h, conversion to general anesthesia with intubation.

### Anesthesia protocols

The patients were matched by sex, age, and weight and received general anesthesia with intubation (*n* = 25), combined spinal-epidural anesthesia (*n* = 25), and general anesthesia with LMA and nerve block (*n* = 25). All patients fasted preoperatively for 8 h. Hypertension and diabetes mellitus were controlled before the surgery. All anesthesia procedures were performed by one anesthesiologist.

For the general anesthesia with intubation, anesthesia was induced by infusing midazolam 0.05 mg/kg, lidocaine 0.5 mg/kg, fentanyl 3 μg/kg, propofol 1.5–2.0 mg/kg, and cisatracurium 0.2 mg/kg. Then the patients were intubated for mechanical ventilation.

For the combined spinal-epidural anesthesia, the patient was in the lateral decubitus position. Ropivacaine 0.5% 4 ml was injected into the subarachnoid space through the L2-L3 intervertebral space. An epidural catheter was positioned. The upper level of anesthesia was controlled bellow the T8 vertebra. Ropivacaine 0.5% 3–5 ml was administered epidurally if the surgery lasted over 2 h.

For the general anesthesia with LMA and nerve block, lumbar plexus-sciatic nerve block was first performed. A 100-mm stimulation needle (D22G, Stimuplex, B. Braun, Germany) was used to deliver electric stimulations at 2 Hz, with a starting current of 1 mA and a pulse time of 0.1 ms. Contraction of the quadriceps femoris and the gastrocnemius in response to a current < 0.3 mA indicated that the injection site had been reached. After confirming no aspiration of blood or cerebrospinal fluid, ropivacaine 0.5% 30 ml was injected respectively for lumbar plexus block and sciatic nerve block. Anesthesia was induced using etomidate 0.1–0.3 mg/kg, vecuronium bromide 0.1 mg/kg, and fentanyl 2–4 μg/kg. Then an LMA was inserted. General anesthesia was maintained using propofol 3–5 mg/h·kg. No postoperative analgesics were used.

### Collection of patient data

Patient data was from a previous clinical trial in which intraoperative heart rate, systolic blood pressure, and diastolic blood pressure were recorded using a ventilator. Hemodynamic changes were evaluated using the maximum variation rate, which was calculated using the formula:

maximum variation rate = (maximum – minimum)/pre − anesthesia value.

Postoperative pain was evaluated at 2 h, 4 h, 6 h, 8 h, and 24 h using the visual analog scale (VAS). The VAS scores range from 0 to 10, with 0 indicating painless and 10 indicating the worst pain imaginable.

Patient cognitive function was assessed preoperatively, postoperative 15 min, 45 min, and 120 min using the Mini-Mental State Examination (MMSE). The MMSE assesses orientation to place and time, calculation, memory, language, reading, writing, and drawing. A reduction of 3 points in the postoperative MMSE score suggested postoperative cognitive dysfunction [6].

Postoperative respiratory infection was tested by bacteria culture using the respiratory secretions collected postoperative 24 h, 48 h, and 72 h.

### Statistical analysis

Continuous data were presented as means and standard deviations. Categorical data were presented as percentages or frequencies. Comparisons were made using the one-way analysis of variance analysis followed by post-hoc analysis or the chi-square test. Ordinal data was compared using the Kruskal-Wallis test. All statistical analyses were performed using the SPSS 18.0 software (SPSS, Chicago, USA). A *P*-value < 0.05 was considered statistically significant.

## Results

A total of 91 patients were eligible for inclusion and 16 patients were excluded for cognitive impairment (*n* = 7), allergy to anesthetic agents (*n* = 4), operation time > 4 h (*n* = 2), conversion to general anesthesia with intubation (*n* = 3). The patients included 33 men and 42 women (age range, 60–90 years). There was no significant difference in sex, age, weight, and ASA physical status between the three groups (Table 1).

**Table 1:** Patient general information

	General anesthesia with intubation (*n* = 25)	Combined spinal-epidural anesthesia (*n* = 25)	General anesthesia with LMA/nerve block (*n* = 25)	*P*
Male, n (%)	12 (48)	10 (40)	11 (44)	0.850
Age, year	74.7 ± 6.8	75.4 ± 6.1	75.5 ± 6.0	0.893
Weight, kg	62.1 ± 7.6	62.3 ± 6.4	64.8 ± 6.6	0.320
ASA physical status, n (%)				0.796
I	3 (12)	3 (12)	2 (8)	
II	21 (84)	20 (80)	21 (84)	
III	1 (4)	2 (8)	2 (8)	
Operation time (min)	61.1 ± 6.7	62.3 ± 6.7	63.8 ± 7.1	0.782
Anesthesia time (time)	85.7 ± 6.8	86.4 ± 7.0	86.6 ± 7.0	0.842
Hospital stay (day)	15.7 ± 2.2	16.4 ± 2.0	14.56 ± 2.02	0.860

*LMA* laryngeal mask airway, *ASA* American Society of Anesthesiologists

The maximum variation rate of heart rate, systolic blood pressure, and diastolic blood pressure differed significantly between the three groups (general anesthesia with intubation > combined spinal-epidural anesthesia > general anesthesia with LMA and nerve block, all *P* < 0.001, Table 2).

**Table 2:** Comparison of intraoperative hemodynamics

Maximum variation rate	General anesthesia with intubation (*n* = 25)	Combined spinal-epidural anesthesia (*n* = 25)	General anesthesia with LMA/nerve block (*n* = 25)	*P*
Heart rate	0.47 (0.44–0.54)^#*^	0.31 (0.30–0.33 ^*^	0.22 (0.21–0.24)	< 0.001
Systolic blood pressure	0.45 (0.39–0.48)^#*^	0.35 (0.32–0.37)^*^	0.23 (0.21–0.26)	< 0.001
Diastolic blood pressure	0.46 (0.41–0.49)^#*^	0.32 (0.30–0.35)^*^	0.25 (0.23–0.29)	< 0.001

*LMA* Laryngeal mask airway. #vs combined spinal-epidural anesthesia, *P* < 0.001; *vs general anesthesia with LMA/nerve block, *P* < 0.001.

The three groups showed no significant difference in preoperative VAS scores. The VAS scores postoperative 2 h, 4 h, 6 h, and 8 h differed significantly between the three groups (general anesthesia with intubation > combined spinal-epidural anesthesia > general anesthesia with LMA and nerve block, all *P* < 0.001, Table 3). The VAS scores at rest and during ambulation postoperative 24 h were significantly lower in the general anesthesia with LMA/nerve block group than the general anesthesia with intubation group and the combined spinal-epidural anesthesia group.

**Table 3:** Evaluation of perioperative pain.

VAS score	General anesthesia with intubation (*n* = 25)	Combined spinal-epidural anesthesia (*n* = 25)	General anesthesia with LMA/nerve block (*n* = 25)	*P*
Preoperative	6.72 ± 0.79	6.84 ± 0.75	6.76 ± 0.72	0.849
Postoperative 2 h	6.96 ± 0.73^#*^	2.52 ± 0.51^*^	1.48 ± 0.51	< 0.001
Postoperative 4 h	6.48 ± 0.65^#*^	2.72 ± 0.46^*^	1.48 ± 0.51	< 0.001
Postoperative 6 h	6.24 ± 0.52^#*^	3.72 ± 0.94^*^	2.36 ± 0.64	< 0.001
Postoperative 8 h	6.04 ± 0.54^#*^	5.44 ± 0.77^*^	2.76 ± 0.44	< 0.001
Postoperative 24 h at rest	5.72 ± 0.54^*^	6.16 ± 0.62^*^	3.40 ± 0.50	< 0.001
Postoperative 24 h during ambulation	7.24 ± 0.44^*^	7.16 ± 0.37^*^	5.00 ± 0.58	< 0.001

*VAS* Visual analog scale, *LMA* Laryngeal mask airway. #vs combined spinal-epidural anesthesia, *P* < 0.001; *vs general anesthesia with LMA/nerve block, *P* < 0.001.

There was no significant difference in preoperative MMSE scores between the three groups. The MMSE scores postoperative 15 min and 45 min differed significantly between the three groups (general anesthesia with intubation < combined spinal-epidural anesthesia < general anesthesia with LMA and nerve block, all *P* < 0.001, Table 4). The MMSE scores postoperative 120 min in the general anesthesia with intubation group were the lowest among the three groups. However, it did not differ significantly between the combined spinal-epidural anesthesia group and the general anesthesia with LMA/nerve block group.

**Table 4:** Assessment of perioperative cognitive function

MMSE score	General anesthesia with intubation (*n* = 25)	Combined spinal-epidural anesthesia (*n* = 25)	General anesthesia with LMA/nerve block (*n* = 25)	*P*
Preoperative	28.56 ± 1.00	28.48 ± 0.77	28.64 ± 0.57	0.780
Postoperative 15 min	16.60 ± 1.35^#*^	22.68 ± 1.07^*^	24.72 ± 0.98	< 0.001
Postoperative 45 min	20.92 ± 1.22^#*^	24.96 ± 1.21^*^	27.48 ± 0.65	< 0.001
Postoperative 120 min	26.20 ± 1.15^#*^	28.20 ± 0.71	28.48 ± 0.59	< 0.001

*MMSE* Mini-Mental State Exam, *LMA* Laryngeal mask airway. #vs combined spinal-epidural anesthesia, *P* < 0.001; *vs general anesthesia with LMA/nerve block, *P* < 0.001.

There was no significant difference in the incidence of respiratory infection postoperative 24 h, 48 h, and 72 h between the three groups (Table 5).

**Table 5:** Perioperative respiratory infection.

	General anesthesia with intubation (*n* = 25)	Combined spinal-epidural anesthesia (*n* = 25)	General anesthesia with LMA/nerve block (*n* = 25)	*P*
Preoperative, n	0	0	0	
Postoperative 24 h, n	0	0	0	
Postoperative 48 h, n	2	1	0	0.769
Postoperative 72 h, n	3	1	0	0.315

*LMA* laryngeal mask airway.

## Discussion

Our study found that general anesthesia with LMA and nerve block was associated with less significant intraoperative hemodynamic variations compared to general anesthesia with intubation and combined spinal-epidural anesthesia. In addition, patients receiving general anesthesia with LMA and nerve block also had significantly less postoperative pain and significantly better postoperative cognitive function than those receiving the other two anesthesia methods.

Hemodynamic instability during intubation and extubation, such as changes in heart rate and blood pressure, can increase the risk of vascular events, especially in elderly patients [7]. In our study, patients receiving the combined spinal-epidural anesthesia also had significantly greater hemodynamic variations compared to those receiving general anesthesia with LMA and nerve block. Spinal anesthesia can inhibit the sympathetic nerves, leading to peripheral vascular dilation and hypotension [8–10]. In addition, vagus nerve dominance and slow heart rate during spinal anesthesia may also result in significant hemodynamic variations. On the contrary, general anesthesia with LMA and nerve block has been shown to have less effect on hemodynamics [5, 11, 12].

Our study found that patients receiving general anesthesia with LMA and nerve block had generally less postoperative pain compared to those receiving general anesthesia with intubation or combined spinal-epidural anesthesia. Spinal anesthesia and nerve block both can effectively stop the peripheral afferent pain pathways and provide good analgesic effect. However, spinal anesthesia was not as good as lumbar plexus/sciatic nerve block in terms of analgesic effect and duration in our study. This might be explained by the relatively small doses of anesthetic agents used in spinal anesthesia, which was resulted from controlling the level of anesthesia.

The MMSE is widely used for screening cognitive dysfunction. It is easy to use and has a sensitivity of 87% and a specificity of 82% in diagnosing postoperative cognitive dysfunction [6, 13–15]. Our study found that general anesthesia with LMA and nerve block was associated with generally better postoperative cognitive function compared to general anesthesia with intubation and combined spinal-epidural anesthesia. In addition, combined spinal-epidural anesthesia was also superior to general anesthesia with intubation in terms of postoperative cognitive function. We speculate that the relatively poor postoperative cognitive function of patients receiving general anesthesia with intubation was associated with the residual systemic analgesics, which might inhibit the central nervous system [16–19]. In addition to analgesics, pain may also contribute to the development of postoperative cognitive dysfunction [20–22]. The relatively less postoperative pain in patients receiving general anesthesia LMA and nerve block might be a reason for the higher MMSE scores in this group of patients.

During postoperative 72 h, there were 3 cases of respiratory infection in the group of general anesthesia with intubation. However, none of the patients had respiratory infection in the general anesthesia with LMA/nerve block group, and only 1 patient had this condition in the combined spinal-epidural anesthesia group. Although no airway device was used in the combined spinal-epidural anesthesia group, still 1 case of respiratory infection occurred in this group. This might be related to the high level of anesthesia and respiratory paralysis. Intratracheal intubation is more invasive than LMA and may increase the risk of respiratory infection. Two meta-analysis showed that LMA is superior to, or as good as, intratracheal intubation regarding respiratory infection [23, 24].

Our study is not without limitations. First, the sample size is small. Second, our study is a single-center study and may lack representativeness. Third, data was collected from a short postoperative period.

In conclusion, general anesthesia with LMA and nerve block was associated with less postoperative pain and less disturbances on intraoperative hemodynamics and postoperative cognitive function for elderly patients undergoing intertrochanteric fracture surgeries. LMA might also be associated with reduced risks of respiratory infection.

## Data Availability

The datasets used and/or analyzed during the current study are available from the corresponding author on reasonable request.

## Abbreviations

ASA: American Society of Anesthesiologists
LMA: Laryngeal mask airway
MMSE: Mini-Mental State Exam
VAS: Visual analog scale
